# Probing the HIV-1 Genomic RNA Trafficking Pathway and Dimerization by Genetic Recombination and Single Virion Analyses

**DOI:** 10.1371/journal.ppat.1000627

**Published:** 2009-10-16

**Authors:** Michael D. Moore, Olga A. Nikolaitchik, Jianbo Chen, Marie-Louise Hammarskjöld, David Rekosh, Wei-Shau Hu

**Affiliations:** 1 HIV Drug Resistance Program, National Cancer Institute, Frederick, Maryland, United States of America; 2 Myles H. Thaler Center for AIDS Research, University of Virginia, Charlottesville, Virginia, United States of America; Duke University Medical Center, United States of America

## Abstract

Once transcribed, the nascent full-length RNA of HIV-1 must travel to the appropriate host cell sites to be translated or to find a partner RNA for copackaging to form newly generated viruses. In this report, we sought to delineate the location where HIV-1 RNA initiates dimerization and the influence of the RNA transport pathway used by the virus on downstream events essential to viral replication. Using a cell-fusion-dependent recombination assay, we demonstrate that the two RNAs destined for copackaging into the same virion select each other mostly within the cytoplasm. Moreover, by manipulating the RNA export element in the viral genome, we show that the export pathway taken is important for the ability of RNA molecules derived from two viruses to interact and be copackaged. These results further illustrate that at the point of dimerization the two main cellular export pathways are partially distinct. Lastly, by providing Gag *in trans*, we have demonstrated that Gag is able to package RNA from either export pathway, irrespective of the transport pathway used by the *gag* mRNA. These findings provide unique insights into the process of RNA export in general, and more specifically, of HIV-1 genomic RNA trafficking.

## Author Summary

High genetic diversity of HIV-1 presents a difficult barrier for drug treatment and vaccine development. HIV-1 efficiently reassorts mutations in its genome by packaging two copies of RNA into one virion and using portions of each RNA as the template for DNA synthesis. Thus, a key to HIV-1's genetic diversity is how it packages two RNAs into one virion. We have previously shown that HIV-1 packages RNA as a dimer, rather than two monomers. In this report, we determined that HIV-1 RNA finds its copackaged dimer partner mostly in the cytoplasm, not the nucleus, of the infected cells. We also found that the host cell machinery used to transport the HIV-1 RNA out of the nucleus affects the ability of two RNAs to copackage into one virion: HIV-1 RNAs transported using the CRM-1 pathway does not copackage efficiently with RNA transported by the NXF1 pathway. This is also the first demonstration that RNAs transported by the two major cellular pathways, CRM-1 and NXF1, are partially segregated at some point in the cytoplasm. Therefore, this work provides novel insights to both HIV replication mechanisms and cellular RNA transport pathways.

## Introduction

HIV-1 full-length RNAs serve at least two functions: as a template for Gag/Gag-Pol translation, and as genetic material packaged in the virion. Many cellular factors ensure the correct macromolecular trafficking between nucleus and cytoplasm; specifically, mechanisms exist to prevent the export of intron-containing transcripts, such as the full-length HIV-1 RNA [1],[2]. Most cellular mRNAs are fully spliced before export and many are believed to exit the nucleus via the NXF1-dependent pathway [3]. However, many proteins and some RNAs use an alternative, CRM-1-dependent pathway to migrate out of the nucleus [3]. The extent to which these two pathways are linked or overlap is currently unknown, and the reason for their differential use is subject to speculation. It is interesting to note that members of the Retroviridae family can use different transport pathways for the export of their intron-containing RNA. Some viruses, such as Mason-Pfizer monkey virus (MPMV), use the NXF1 pathway by binding the NXF1 protein directly to its RNA via a structured motif (constitutive transport element or CTE) [4]. Other viruses, such as HIV-1, use the CRM-1 pathway by indirectly linking the CRM-1 protein to the viral RNA via a virally encoded protein. In HIV, the viral protein is Rev and it acts as a bridge between a structured RNA motif (the Rev-response element (RRE)) and the CRM-1 protein [5],[6]. Additionally, some retroviruses use pathways that have yet to be identified [7].

Many studies have shown that it is possible to alter the retroviral RNA transport pathway by manipulating the cis- and trans-acting viral elements. Interestingly, a recent study indicates that the transport pathway the RNA takes can influence the function of the protein it encodes. By altering the transport pathway of the HIV-1 Gag-encoding RNA from CRM-1 to NXF1, one can relieve the HIV-1 assembly block in murine cells [8]. Intriguingly, Gag was expressed efficiently in murine cells by RNA using either pathway. However, efficient assembly only occurred when Gag was translated from RNA using the NXF1 pathway; Gag translated from the CRM-1 pathway was mistargeted and could not assemble efficiently [9]. These studies revealed that the transport pathway may play a far more intricate role during viral replication than we previously realized.

Retroviruses package two copies of full-length RNA in one virion. One of the consequences of packaging two RNAs is frequent recombination during DNA synthesis when reverse transcriptase uses parts of both RNAs as templates [10],[11]. Although frequent recombination can occur during DNA synthesis of all virions, a genotypically different recombinant can only be generated from virions that package two different RNAs (heterozygous virions) [10]. Using a recombination assay, we have shown that RNA molecules derived from two similar HIV-1 proviruses can randomly assort and be efficiently copackaged into virions [12],[13]. However, heterozygous virions are formed less efficiently when the two proviruses contain variations in their dimerization initiation signal (DIS). Located at the loop of stem-loop 1 of the 5′ untranslated region, the DIS is a 6-nt palindromic sequence that forms the initial interaction between the two HIV-1 RNAs [14]. The Gag polyproteins of HIV-1 interact with, and specifically package, the viral RNA to generate infectious viruses. We have previously examined whether RNA dimerization occurs prior to virus assembly using HIV-1 variants with DIS mutations that abolish their palindromic nature (for example, from GCGCGC to GGGGGG) but can form perfect base pairs with the DIS of a partner virus (such as a virus with CCCCCC at the DIS). We reasoned that in the coinfected cells if dimeric RNAs are packaged, then the GGGGGG viral RNA would preferentially pair with CCCCCC viral RNA, and we would therefore observe an increase in the formation of heterozygous virions. In contrast, if two monomeric RNAs are packaged, then we would not observe an increase in heterozygous viruses. Our results revealed that most of the virions from coinfected cells were heterozygous, indicating that copackaged RNA partner selection, i.e. dimerization, occurred prior to the packaging of virion RNA [15].

Many questions remain concerning how HIV-1 full-length RNA traffics through the cell to fulfill its various roles. For example, it has been unclear at which point along the RNA trafficking pathway, from the site of transcription to the site of viral budding, HIV-1 RNA selects its copackaged RNA to initiate the dimerization process. It remained unclear whether HIV-1 RNAs are dimerized in the nucleus and traffic out of the nucleus as a dimer, or exit the nucleus as a monomer and dimerize in the cytoplasm. Furthermore, it is not known whether the RNA transport pathway used by the viral RNA affects its interactions with other viral RNA or Gag proteins, and thereby influences the abilities of the RNA to dimerize and be packaged into a nascent virion. In this current work, we addressed these questions by using three systems: a cell-fusion-dependent recombination assay to establish the cellular location of the dimerization event, a Rev-independent HIV-1 to investigate the influence of the NXF1 and CRM-1 export pathways on these processes, and a single virion visualization assay to directly analyze the RNA content of each viral particle. These studies resulted in three novel findings. We demonstrate that the majority of HIV-1 RNA dimerization occurs within the cytoplasm, not within the nucleus. Moreover, the RNA transport pathway taken by the viral RNA has a major effect on the ability of the RNA to be copackaged; these results revealed that RNAs trafficking through the CRM-1 and NXF1 transport pathways are partially separated at least at the stage of RNA commitment to dimerization. Finally, we demonstrate that although the RNA pathways are partially distinct at the point when initial dimerization occurs, selection of packaged RNA by Gag does not discriminate between RNA in these two pathways. These findings provide insights into the mechanisms of HIV replication including RNA-RNA and RNA-Gag interactions, and demonstrate a new approach to the unraveling of the detailed mechanisms of the cell's nuclear-cytoplasmic trafficking pathways.

## Results

### The Cellular Location of HIV-1 RNA Partner Selection and Initial Dimerization

**Figure 1:**
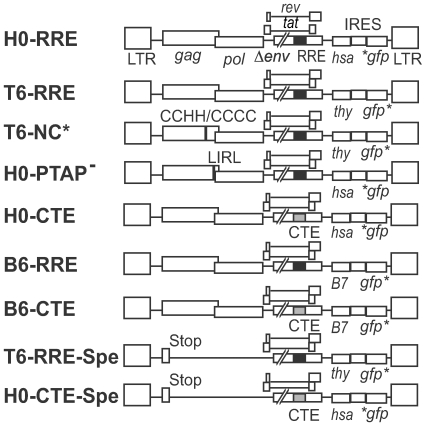
General structures of the viruses used in the recombination and packaging assays. H0-RRE is shown on top and elements of other viruses different from H0-RRE are labelled. Two marker genes are located in *nef* of each virus; the translation of the *gfp* gene was directed by IRES. The inactivating mutations in *gfp* are shown as asterisks. Black box represents the HIV-1 RRE, whereas gray box represents four copies of MPMV CTE.

Our previous experiment revealed that the selection of the copackaged RNA occurs prior to encapsidation into the virion [15]. In the current study, we sought to determine the subcellular location where RNA-RNA interactions take place to initiate the dimerization process. To do this, we developed a cell fusion-based recombination assay to examine whether RNA partner selection is initiated in the cytoplasm. Our previously established recombination assay uses dual-infected cell lines to provide accurate and reproducible measurements of recombination between two viruses [13]. In this system, the near-full-length HIV-1 genome encodes two markers in *nef*, an inactivated green fluorescent protein gene (*gfp*) and a second gene that is either a mouse heat-stable antigen gene (*hsa*) or a mouse thy1.2 gene (*thy*) (Fig. 1). The two inactivating mutations of *gfp* in these two viruses are separated by a distance of 600 bp. A proportion of the viral particles released from the dual-infected cell lines contain one RNA from each provirus (heterozygous viruses); recombination between which can occur during reverse transcription to generate a recombinant progeny expressing a functional *gfp*. The multiplicity of infection (MOI) of the GFP^+^ events, as a percentage of the total infection MOI (determined using the expression of HSA and Thy), provides a measure of the recombination efficiency between the two viruses. As retroviral recombination occurs between two copackaged RNAs, the rate of recombination is dependent on the formation of heterozygous virions; therefore, recombination can be used as a measurement of the dimerization efficiency between two viral RNAs, as we have previously shown [15].

**Figure 2:**
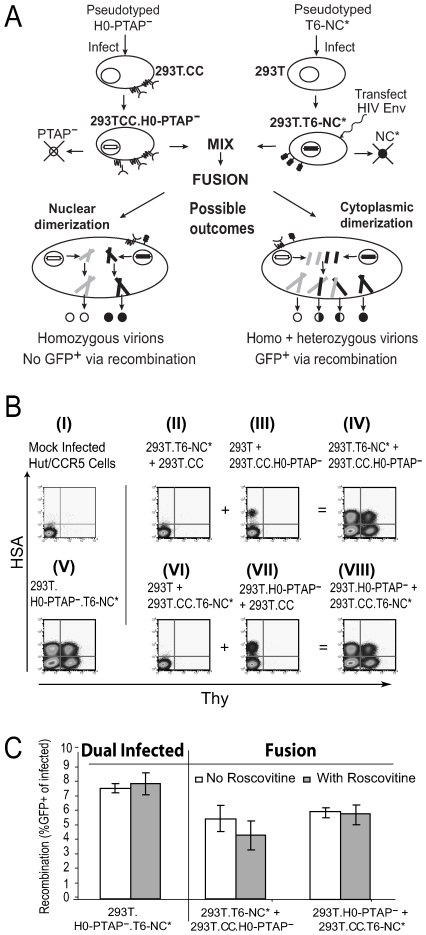
The fusion-dependent recombination assay. (A) An example of the fusion experiment, with predicted outcomes, is shown schematically using the H0-PTAP^–^-infected 293T.CC cell line (293T.CC.H0-PTAP^–^) and a T6-NC*-infected 293T cell line (293T.T6-NC*). The reciprocal experiment using 293T.CC.T6-NC* and 293T.H0-PTAP^–^ was also performed. (B) Representative flow cytometry analyses of target cells infected with viruses harvested from various fusion experiments. The cells used in the fusion experiments, from which viruses were harvested, are indicated above each flow cytometry plot; all 293T cells were transiently transfected with plasmid expressing HIV-1 Env. The x- and y-axis show Thy and HSA expression, respectively. Panel I, mock infection; panel V, positive control, viruses harvested from a producer cell line dual-infected with H0-PTAP^–^ and T6-NC*. Panels II, III, VI, and VII, viruses harvested from fusing one cell line containing a mutant provirus with another cell line that does not contain any HIV-1 provirus; panels IV and VIII, viruses harvested from fusing a T6-NC*-containing cell line with an H0-PTAP^–^-containing cell line. (C) Observed recombination rates from the fusion-based recombination assay with (in gray) or without (in white) the drug roscovitine. Recombination rates generated using viruses harvested from dual-infected cells are shown for comparison. Means and standard deviations generated from three experiments are shown.

We modified the recombination system to examine whether we could detect cytoplasmic RNA dimerization events (Fig. 2). In this system, two cell lines are generated, each infected with a *gag* mutant virus that has a severe replication defect. When the two cell lines are fused using conditions that facilitate cytoplasmic but not nuclear fusion, the Gag proteins from these two viruses can coassemble and complement each other's function to rescue virus replication. Our previous study demonstrated that HIV-1 RNA dimerization occurs prior to Gag packaging [15], and we envision two differential outcomes that would be reflective of the locations of the dimerization event (Fig. 2A). If the selection of the copackaged RNA mostly occurs in the nucleus, the majority of the viruses generated from this system will contain two RNAs derived from the same provirus (homozygous virions) that have the same inactivating *gfp* mutation. Therefore, very few GFP^+^ phenotypes will be formed by recombination. In contrast, if the selection of the copackaged RNA mostly occurs in the cytoplasm, fusing the cytoplasm of the two cell lines will allow the RNAs derived from the two nuclei to interact and dimerize prior to packaging by Gag. In this scenario, heterozygous virions can be formed, which can generate numerous GFP^+^ recombinants. Therefore, in the fusion experiment, the location of the RNA dimerization event, in the nucleus or in the cytoplasm, predicts different levels of the recombinant GFP^+^ phenotype.

We generated cell lines containing either a nucleocapsid (NC) mutant virus (T6-NC*) or a late domain mutant virus (H0-PTAP^–^). The NC* mutation reduces RNA packaging to 5% of wild-type and abolishes infectivity [16] (Fig. 2B panels II and VI), whereas the PTAP^–^ mutation reduces viral budding and viral titer drops to ∼2% of wild type [17],[18] (Fig. 2B panels III and VII). To facilitate fusion, one cell line was based on modified 293T cells that express CD4 and CCR5 (293T.CC), whereas the other cell line was 293T based and was transiently transfected, prior to fusion, with a plasmid expressing CCR5-tropic HIV-1 Env. Viruses were harvested 16 h after the coculture of the two cell lines; at this time point, cell fusion but not nuclear fusion was visible by microscopy (Fig. S1 and data not shown). Target cells were infected with these viruses, and flow cytometry was performed to measure virus infection and recombination. Our results (Fig. 2C) demonstrate that the recombination rates of infectious viruses produced by cell fusion (5–6%) are similar to that of our standard recombination assay (7%), which uses viruses produced from cells containing two proviruses [13]. To confirm that nuclear fusion did not affect the results of this assay, the cyclin-dependent kinase inhibitor roscovitine was added after mixing the cell lines. Roscovitine was previously shown, in a detailed study of HIV-1-directed cell fusion, to prevent nuclear envelope breakdown and nuclear fusion or karyogamy [19]. We observed similar recombination rates in samples with or without roscovitine treatment (Fig. 2C), indicating that nuclear fusion was not a factor in the high levels of recombination detected. Taken together, these results indicate that most of the RNA dimerization and partner selection process occurs in the cytoplasm of the producer cells.

### The Impact of the RNA Export Pathway on Recombination

Having demonstrated that the majority of HIV-1 RNA dimerization occurs within the cytoplasm, we further investigated whether the pathway of entry into the cytoplasm is important for RNA dimerization and packaging. To this end, we generated two HIV-1 variants, H0-CTE and B6-CTE, that use the NXF1, and not the CRM-1, transport pathway by removing the RRE motif and replacing it with four copies of the MPMV CTE [20]. H0-CTE encodes the same HSA and mutated *gfp* marker as H0-RRE (Fig. 1); B6-CTE encodes a mouse B7 gene and a *gfp* that has the inactivating mutation located at the 3′ end of the gene (Fig. 1). Using positive control viruses with two functional genes, we have previously showed that the HSA and GFP or Thy and GFP markers are well-expressed and can be simultaneously detected efficiently by flow cytometry [13]; we have also examined the B7 and GFP expression and showed these two markers were detected simultaneously and efficiently by flow cytometry (Fig. S2).

**Figure 3:**
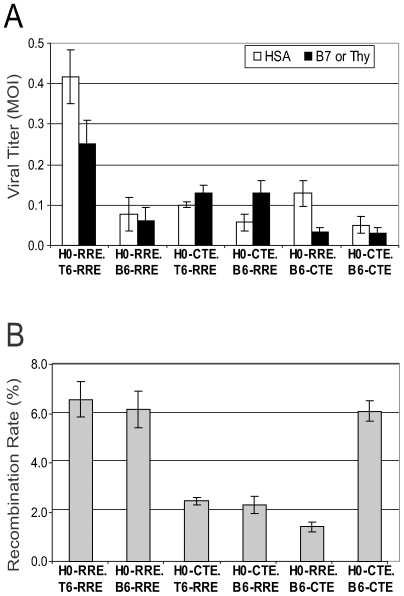
Effect of nuclear export pathway on recombination. (A) Titers of the HSA and Thy, or HSA and B7 viruses generated from producer cell lines. White bars represent titers of viruses encoding HSA, whereas black bars represent titers of viruses encoding Thy or B7, respectively. (B) Observed recombination rates using viruses from each cell line. Means and standard deviations generated from three experiments are shown.

To assess the effect of the nuclear export pathway on HIV-1 RNA dimerization and copackaging, six types of dual-infected cell lines were generated containing either two RRE-dependent proviruses (H0-RRE.T6-RRE; H0-RRE.B6-RRE), two CTE-dependent proviruses (H0-CTE.B6-CTE), or one RRE- and one CTE-dependent provirus (H0-CTE.T6-RRE, H0-CTE.B6-RRE, and H0-RRE.B6-CTE). Flow cytometry analyses of the markers encoded by the two viruses indicated that most of the cells (>90%) in each cell line express both viruses (data not shown). These cell lines were transiently transfected with a plasmid expressing HIV-1 Env and the released viruses were used to infect target cells; the titers generated from these infections are shown in Fig. 3A. In general, the two proviruses in each cell line generated titers within two fold of each other; the only notable exception was H0-RRE.B6-CTE, from which the B6-CTE titer was much lower than that of H0-RRE. We also measured the recombination rates generated from these cell lines; results from three experiments are summarized in Fig. 3B. When two viruses both used the CRM-1 pathway (H0-RRE.T6-RRE and H0-RRE.B6-RRE), recombination rates were 6.2–6.6%, similar to the previously measured 7% rate using the H0-RRE.T6-RRE cell line. When both viruses used the NXF1 pathway (H0-CTE.B6-CTE), the recombination rate was similar to that of the two RRE viruses; these results indicated that HIV-1 RNAs from two proviruses both using the NXF1 export pathway can copackage together efficiently. In contrast, recombination between a CTE-using and an RRE-using HIV-1 is lower than expected from random mixing of the viral genomes. The two viruses from H0-CTE.T6-RRE cell lines have similar titers, and if their RNAs were copackaged together randomly, we would have expected a 6–7% recombination rate; however only 2.5% of the viruses generated GFP^+^ recombinants. Similar low rates of recombination were also observed in viruses harvested from H0-CTE.B6-RRE cell line and H0-RRE.B6-CTE cell line. Therefore, HIV-1 variants separately transported by CRM-1 and NXF1 pathways recombined less frequently than two variants using the same pathway, either by CRM-1 or by NXF1. These results suggest that HIV-1 RNAs exported by the CRM-1 and NXF1 pathways are not copackaged together efficiently.

### Single Virion Analyses of Copackaged HIV-1 RNA Molecules Transported by CRM-1 or NXF1 Pathways

**Figure 4:**
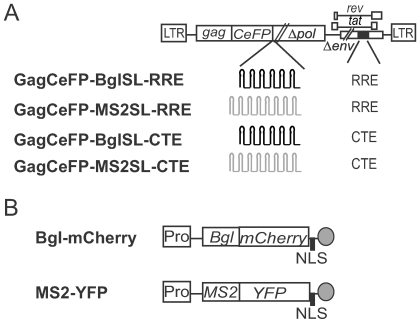
General structures of the plasmids used for single virion visualization. (A) Modified HIV-1 genomes used in the study. Abbreviations are the same as Fig. 1. Stem loop sequences recognized by BglG and MS2 coat proteins are shown in black and grey, respectively; 18 Bgl stem loops and 24 MS2 stem loops are used in these constructs although fewer stem loops are shown. (B) RNA binding proteins tagged with fluorescent proteins used in the study. Pro, Pol II promoter, NLS, nuclear localization signal, grey circle, transcription termination signal.

To directly examine whether RNA exiting the nucleus by different RNA transport pathways can be copackaged together efficiently, we analyzed the RNA content of the viral particles. Utilizing the specific interactions between RNA binding proteins and RNA secondary structures, we recently developed a method to simultaneously detect two RNA species in virion at single RNA molecule sensitivity [21]. This system uses modified HIV-1 genomes that contain the stem-loop sequences, referred to as MS2SL and BglSL, which are recognized by bacteriophage MS2 coat protein and *E. coli* antitermination protein BglG, respectively (Fig. 4A). These HIV-1 genomes also express Gag or a Gag-cerulean fluorescent protein (CeFP) fusion protein. When these HIV-1 genomes are coexpressed with the MS2 coat protein fused to a yellow fluorescent protein (MS2-YFP), or the N-terminal fragment of BglG fused to a red fluorescent protein (Bgl-mCherry) (Fig. 4B), the presence of viral RNA can be visualized by the fluorescent signals. To analyze the virion RNA, plasmids expressing the MS2SL- or BglSL-containing HIV-1 genomes were cotransfected with plasmids expressing MS2-YFP or Bgl-mCherry. Viral particles were harvested and visualized; representative images are shown in Fig. S3 and summarized in Table 1. In these experiments, HIV-1 particles are detected by CeFP signals, whereas RNA molecules are detected by YFP or mCherry signals. It has been shown that when coexpressed with wild-type Gag, Gag-GFP fusion protein can coassemble into virus-like particles indistinguishable from wild-type immature particles [22],[23]. Thus, in all RNA visualization experiments, a pair of nearly-identical HIV-1 constructs were used, one expressing Gag-CeFP and the other expressing wild-type Gag; for concise description of these experiments, only the name of the CeFP-tagged construct is used in the text.

**Table 1:** RNA contents in viral particles by Single virion visualization.

Group	Construct tranfected		CeFP^+^ particles analyzed	% CeFP^+^ & YFP^+^	% CeFP^+^ & mCherry^+^	% CeFP^+^ & YFP^+^ & mCherry^+^	RNA Labeling efficiency*
1	GagCeFP-BglSL-RRE + Bgl-mCherry						
		Exp.a	1668	0.0	97.7	0.0	97.7
		Exp.b	931	0.0	95.7	0.1	95.8
2	GagCeFP-MS2SL-RRE + MS2-YFP						
		Exp.a	2042	93.0	0.0	0.0	93.0
		Exp.b	1058	96.1	0.0	0.2	96.3
3	GagCeFP-BglSL-CTE + Bgl-mCherry						
		Exp.a	1193	0.0	92.6	0.0	92.6
		Exp.b	479	0.0	90.8	0.2	91.0
4	GagCeFP-MS2SL-CTE + MS2-YFP						
		Exp.a	1540	90.5	0.0	0.0	90.5
		Exp.b	533	92.9	0.0	0.2	93.1
5	GagCeFP-BglSL-RRE + MS2-YFP						
		Exp.a	664	0.3	0.0	0.0	0.3
		Exp.b	1260	0.2	0.0	0.0	0.2
6	GagCeFP-MS2SL-RRE + Bgl-mCherry						
		Exp.a	410	0.0	0.0	0.0	0.0
		Exp.b	649	0.0	0.0	0.0	0.0
		Exp.c	304	0.0	0.0	0.0	0.0
7	GagCeFP-BglSL-CTE + MS2-YFP						
		Exp.a	633	0.5	0.0	0.0	0.5
		Exp.b	239	0.0	0.0	0.0	0.0
		Exp.c	368	0.8	0.0	0.0	0.8
8	GagCeFP-MS2SL-CTE + Bgl-mCherry						
		Exp.a	436	0.2	0.0	0.0	0.2
		Exp.b	419	0.2	0.0	0.0	0.2
9	GagCeFP-BglSL-RRE + GagCeFP-MS2SL-RRE + Bgl-mCherry + MS2-YFP						
		Exp.a	962	28.3	18.0	50.7	97.0
		Exp.b	986	22.4	21.8	47.1	91.3
10	GagCeFP-BglSL-CTE + GagCeFP-MS2SL-CTE + Bgl-mCherry + MS2-YFP						
		Exp.a	763	32.0	21.5	39.8	93.3
		Exp.b	488	25.4	23.2	44.1	92.6
11	GagCeFP-BglSL-RRE + GagCeFP-MS2SL-CTE + Bgl-mCherry + MS2-YFP						
		Exp.a	713	48.1	21.3	21.6	91.0
		Exp.b	517	33.8	39.1	22.2	95.2
12	GagCeFP-BglSL-CTE + GagCeFP-MS2SL-RRE + Bgl-mCherry + MS2-YFP						
		Exp.a	648	40.1	32.6	22.2	94.9
		Exp.b	552	37.0	33.7	22.6	93.3

*: RNA labeling efficiency is the sum of the three previous columns (%CeFP^+^ & YFP^+^; % CeFP^+^ & mCherry^+^ ; % CeFP^+^ & YFP^+^ & mCherry^+^).

In agreement with our previous results [21], when Bgl-mCherry was coexpressed with GagCeFP-BglSL-RRE, most of the CeFP^+^ particles also have the mCherry signal (>96%; Table 1, Group 1). The mCherry signal is specific to the BglSL in the viral RNA genome; particles derived from HIV-1 with similar structures but containing MS2SL did not have mCherry signals when coexpressed with Bgl-mCherry (Table 1, Group 5). Similarly, MS2-YFP also specifically labeled the HIV-1 genome with MS2SL at high efficiency (∼95%) but not HIV-1 RNA with Bgl SL (Table 1, Group 2 and 6, respectively). When we examined particles derived from HIV-1 genomes using the CTE for export, we observed similar RNA labeling efficiency by the Bgl-mCherry and MS2-YFP proteins (both ∼92%; Table 1, Group 3 and 4).

Examination of viral particles generated from coexpression of the Bgl and MS2 systems can determine the distribution of the viral genomes at a single RNA level [21]. When both BglSL and MS2SL containing HIV-1 genomes used the RRE element for RNA transport, these two RNA species were copackaged together efficiently (Table 1, Group 9); ∼49% of the CeFP^+^ particles have both the mCherry and YFP signals (expected to be 50% by Hardy-Weinberg equilibrium). Similarly, when both HIV-1 genomes used the CTE element for RNA export, these RNA species were also copackaged together efficiently; ∼42% of the CeFP^+^ particles have both RNA signals (Table 1, Group 10). This finding agrees with our recombination rate studies that efficient copackaging of HIV-1 RNA is not dependent on the CRM-1 transport pathway; two RNA species transported by the NXF1 pathway can also copackage together efficiently.

We then examined particles generated from coexpression of HIV-1 genomes containing RRE or CTE. As shown in Table 1 (Group 11 and 12), RRE-containing HIV-1 RNA did not copackage efficiently with CTE-containing HIV-1 RNA; most of the CeFP^+^ particles were labeled with one RNA signal, either mCherry or YFP, and only ∼22% of the CeFP^+^ particles were labeled with both YFP and mCherry signals regardless of whether the RRE-containing RNA was detected by MS2-YFP or by Bgl-mCherry. These results indicate that the RRE- and CTE-containing RNAs were not copackaged together as frequently as two RRE-containing RNAs or two CTE-containing RNAs. These findings are in agreement with our data demonstrating a decreased recombination rate between RRE- and CTE-containing HIV-1 variants (Fig. 3B).

Taken together, our results show that HIV-1 RNAs exported by the CRM-1 and NXF1 pathways are not copackaged together efficiently; therefore, RNA dimerization occurs at a point where RNAs from the two pathways are partially segregated and not completely mixed. However, both the recombination assay and single virion analyses revealed that the RNAs transported by the different pathways can still be copackaged, albeit less efficiently, indicating a partial overlap of the RNAs from the two pathways at the time of RNA dimerization and packaging.

### The Effect of Nuclear Export Pathways on RNA Packaging by Gag

**Figure 5:**
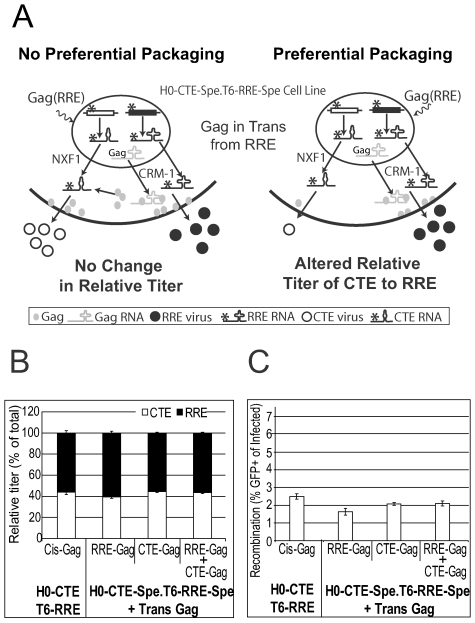
Effect of nuclear export pathway used by *gag* mRNA on genomic RNA packaging and recombination. (A) Schematic representations of the expected outcomes when Gag is provided in trans by mRNA using the CRM-1 pathway. (B) Proportion of the two viruses released from the H0-CTE-Spe.T6-RRE-Spe cell line when Gag is provided in trans by mRNA using either the CRM-1 or NXF1 pathway, or both. The virus proportions when Gag is provided *in cis* are also shown for comparison. (C) The recombination rate between the two viruses when Gag is provided in trans. Means and standard deviation generated from three experiments are shown.

The transport pathway used by the RNA encoding HIV-1 Gag has been shown to affect the downstream assembly events in rodent cells, indicating a link between RNA trafficking and protein function [8],[9]. Our results showed that RNAs using different export pathways are not copackaged randomly; however, it was unclear whether the use of different RNA export pathways also affects the interaction between the RNA and viral Gag proteins, thereby affecting the selection of packaged RNAs. If the RNA export pathway dictates the location of Gag translation and nascent Gag molecules favor the packaging of RNA within the same locality, then Gag would be expected to demonstrate a preference for packaging viral RNA exported via the same pathway as that of the *gag* mRNA (Fig. 5A). Conversely, Gag could demonstrate no preference for selecting and packaging viral RNA exported by different pathways, irrespective of the transport pathway used by its own mRNA.

To explore the impact of the RNA export pathway used by *gag* mRNA, we generated a cell line infected with two viruses, H0-CTE-Spe and T6-RRE-Spe (Fig. 1). Each of these viruses used a different RNA transport pathway and contained an inactivating frameshift mutation in *gag*. We then provided Gag *in trans* by transfection of the cell line with various Gag-Pol expression plasmids. The Gag-encoding mRNA is exported from the nucleus by the CRM-1 pathway (RRE-Gag), the NXF1 pathway (CTE-Gag), or both pathways (RRE-Gag and CTE-Gag). We then compared the relative viral titers generated from these experiments with those from the H0-CTE.T6-RRE cell line, in which both viruses expressed functional Gag and the relative viral titers were 44%:56% (HSA: Thy; 1^st^ lane Fig. 5B). If preferential packaging occurred, we would expect to see an alteration in the relative viral titers of the HSA and Thy viruses, with the direction of the shift dependent on the export element used to provide Gag. Alternatively, efficient trans-packaging across RNA transport pathways would result in no alteration in the ratios of HSA:Thy released from the H0-CTE-Spe.T6-RRE-Spe cell line.

Our results, summarized from three independent experiments, demonstrate that the same relative levels of the two viruses (H0-CTE-Spe and T6-RRE-Spe) were detected, irrespective of the RNA export pathway used by the *gag* mRNA (Fig. 5B). Similar results were obtained by infecting this cell line with a virus expressing functional Gag (data not shown). Therefore, Gag packages viral genomes with similar efficiency regardless of the RNA transport pathways used by the full-length viral RNA or the Gag mRNA. This observation further extends our previous findings, demonstrating the ability of Gag to efficiently package RNA in trans [24]. Moreover, we also observed that the resultant recombination between the two Gag mutant viruses was unaffected by the RNA transport pathway used by the supplied Gag (Fig. 5C), indicating that the proportion of heterozygous virions in the viral population was not altered. These results are consistent with our previous observation that copackaged RNA selection and dimerization occur prior to encapsidation into the virion.

## Discussion

The diverse roles played by the full-length HIV-1 RNA necessitate strict control over each aspect of its biogenesis and fate. Initially, newly synthesized RNA must be spliced for expression of early gene products such as Rev and Tat. Once accumulated to sufficient levels, Rev directs the export of the RRE-containing transcripts, including the unspliced RNA. Once in the cytoplasm, full-length viral RNA can be used for Gag/Gag-Pol translation, or can be packaged into nascent particles as the template for DNA synthesis in the next replication cycle. At least two viral proteins, Rev and Gag, are involved in the trafficking of the full-length RNA from the nucleus to the viral particle. Additionally, interactions between the viral components and the cellular pathways play a crucial role in the process, the extent and specifics of which are not yet completely understood. Our findings in this work provide an outline of events that lead to packaging of the HIV-1 full-length RNAs. Following export from the nucleus, HIV-1 full-length RNA selects its copackaged RNA partner by forming base-pairing at the DIS. Our data demonstrated that the initial dimerization event occurs in the cytoplasm, at a point where RNA molecules exported from NXF1 and CRM-1 pathways are not fully mixed. Although HIV-1 normally uses the CRM-1 transport pathway, this is not a prerequisite for efficient copackaging of RNAs from two viruses; HIV-1 RNAs derived from two viruses both using the NXF1 pathway for RNA transport can also be efficiently copackaged. The dimeric RNAs then interact with and are packaged by the Gag polyproteins; furthermore, Gag expressed *in trans* can efficiently package HIV-1RNAs exported using either the CRM-1 or the NXF1 pathway. In addition to gaining an increased understanding of the HIV-1 replication cycle, our demonstration that RNAs from the NXF1 and CRM-1 pathways are partially segregated in specific cellular locations also provides insights into the nuclear trafficking pathways themselves.

Our finding that selection of HIV-1 copackaged RNAs and initial dimerization events mainly occur in the cytoplasm provide a contrast to the suggested nuclear MLV dimerization events [25]–[27]. The difference between these two viruses in the location of their RNA dimerization event provides a possible explanation for the stark difference in their respective recombination potentials. We have directly compared the recombination rates of HIV-1 and MLV using the same target sequences separated by 1 kb [13],[28]; the HIV-1 rate under these conditions is 42.4%, whereas the MLV rate is 10-fold lower at 4.7%, even though the reverse transcriptase complex of each virus has a similar capacity to switch templates during DNA synthesis [29]–[31]. As the initial dimerization event of HIV-1 occurs in the cytoplasm, the RNAs from two different HIV-1 proviruses have a great opportunity to mix fully prior to copackaging, thereby allowing efficient formation of heterozygous virions and a high recombination potential. In contrast, MLV appears to have a limited ability to copackage RNAs generated from two separate proviruses. Although frequent crossovers can be observed in MLV recombinants, only a limited number of viruses are capable of generating phenotypically different recombinants. If, as suggested, MLV RNA dimerizes within the nucleus, particularly if it occurs at the site of transcription, then the RNA from two proviruses would be unable to fully mix and heterozygous virions would not be formed efficiently, reducing the recombination potential of the viral population. Hence, the location of the initial dimerization event may directly influence the recombination potential and the ability of the virus to generate variation and evolve. It is curious that two viruses from the same virus family appear to initiate the dimerization events at rather different locations. Interestingly, HIV-1 and MLV may also differ in whether separate pools of RNAs are used for translation and RNA packaging. Elegant early studies have suggested that MLV uses two different pools of full-length RNAs, one for translation of Gag/Gag-Pol and the other for encapsidation into newly synthesized particles [32]. Such separation of RNA pools is not apparent for HIV-1 [33],[34]. We speculate that these differences may reflect distinct strategies used by MLV and HIV-1 to regulate the function of the full-length RNA. Experimental evidence supports that both HIV-1 and MLV Gag package RNA dimers. Therefore, both viruses have to face the dilemma of incompatibility between dimer formation and ribosomal scanning through the dimeric region during translation. We speculate that MLV solves this issue by using early nuclear dimerization events to separate the two RNA pools such that the packaged RNA is dimeric and the translated RNA is monomeric. In contrast, HIV-1 adopts a different strategy, having one pool of full-length RNA capable of translation, dimerization and packaging [33],[34]. Although the exact determinants of the fate of each RNA are unclear, it is probable that dimerization within the cytoplasm plays a key role in distinguishing RNA used for translation from RNA packaged into virions. Both of these proposed strategies bypass the conundrum of translating through dimerized regions and maintain the dual role of the full-length genomic RNA.

In order to transport the full-length or singly spliced RNAs from the nucleus to the cytoplasm, HIV-1 uses the cellular protein-shuttling CRM-1 pathway instead of the NXF1-dependent pathway. To determine the amount of overlap between these two pathways and investigate the reliance of HIV-1 on its particular pathway to assort RNAs transcribed from different proviruses, we generated an RRE-independent HIV-1 that uses the MPMV CTE and the NXF1 pathway for RNA export from the nucleus. Using these viruses, we showed that the ability of RNA from different HIV-1 proviruses to fully assort does not depend on using the CRM-1 pathway. Furthermore, we showed that the two pathways (CRM-1 and NXF1) are distinct at the point of HIV-1 dimerization, although some recombination was detected, indicating a partial overlap. To our knowledge, this is the first time that an interaction between RNAs in these two pathways has been addressed. The partial overlap between the CRM-1 and NXF1 pathways also raises the interesting possibility of RNA-RNA interactions between two differentially regulated viruses, such as simian immunodeficiency virus and MPMV, during the nuclear export of their full-length RNAs within dual-infected cells.

Although the results presented here, which were generated from three different cell lines, clearly and consistently indicate that the recombination rates between an RRE- and a CTE-dependent virus are lower than those from two viruses using the same RNA transport pathway, it is worth noting that this lower recombination rate was not consistently observed when viruses were produced by cotransfection of an RRE- and a CTE-dependent viral DNA. Viral titers generated by our transfection experiments are routinely much higher than those from cell lines containing integrated proviruses, most likely because high expression from multiple copies of transfected DNA. We hypothesize that overexpression in the transiently transfected cells can overload the system and blur the boundaries between the two transport pathways. We believe the results from the cell lines containing integrated proviruses most closely mimic the events during HIV-1 replication in the human population and are more biologically relevant than any transient transfection data.

Using an HIV-1-based minimal vector and a helper construct, it was recently shown that in the absence of the Rev-RRE interaction, the level of vector RNA in the cytoplasm was slightly reduced, but the virion RNA detected in the supernatant was severely affected [35]. Therefore, the RRE-Rev interaction is important for efficient RNA packaging. One possible explanation is that cytoplasmic viral RNA was mistargeted in the absence of functional RRE-Rev interaction and was unable to be efficiently packaged by Gag. In our single virion analyses, the viral particles derived from CTE-using HIV-1 contained viral RNA at similar levels to those derived from RRE-using viruses (Table 1). Additionally, we show that RRE- or CTE-containing RNA can be packaged regardless of the RNA export pathway used by the Gag-expressing RNA (Fig. 5). Together, these findings further support the view that the RRE- or CTE-mediated RNA transport pathway not only exports the RNA from the nucleus but also targets these RNAs to proper compartments where downstream events necessary for viral replication can occur.

Why some retroviruses, such as HIV-1, have evolved a dependence on the CRM-1 pathway whereas other retroviruses, such as MPMV, use the NXF1 pathway remains a mystery. However, the use of two differentially exported HIV-1 viruses within the same coinfected cell line provides a unique tool to analyze this and many other questions. Moreover, the two pathways could be further dissected to define novel cellular factors involved in RNA trafficking, which may lead to the identification of novel targets for the development of antiviral compounds specific for HIV-1 RNA export. This cell line might also provide a basis for a high-throughput screen to identify antiviral compounds that specifically target the interplay of HIV-1 RNA with the CRM-1 pathway.

## Materials and Methods

### Viral Constructs

For clarity, previously described pON-H0 and pON-T6 [13] are referred to as H0-RRE and T6-RRE, respectively. T6-NC* and H0-PTAP^–^ contain a CCHC/CCHC-to-CCHH/CCCC mutation in NC and a PTAP-to-LIRL mutation in p6, respectively. H0-ΔRRE was derived from H0-RRE by deleting the entire RRE (nt 7644–8330 in NL4-3). H0-CTE was generated by inserting a DNA fragment containing four copies of the MPMV CTE (4XCTE) obtained from pGPV-CTEx4 [8] into H0-ΔRRE at the location of the previously removed RRE. B6-RRE was derived from T6-RRE by replacing *thy* with mouse B7 gene; B6-CTE was generated by replacing the NotI-to-XhoI fragment of H0-CTE with that of B6-RRE. T6-RRE-Spe and H0-CTE-Spe each contains a premature stop in *gag* located in the middle of the CA domain. RNA derived from H0-Spe, which has identical structure as H0-RRE except containing the aforementioned premature *gag* stop codon mutation were packaged at a similar efficiency to RNA derived from H0-RRE [24].

Plasmid MS2-YFP was a gift from Robert Singer [36]; Bgl-mCherry has been recently described [21]. For clarity, the previously described GagCeFP-MS2SL and GagCeFP-BglSL [21] are referred to as GagCeFP-MS2SL-RRE and GagCeFP-BglSL-RRE, respectively. These modified NL43-based HIV-1 genomes encode functional *gag*, *tat* and *rev*, and *cis*-acting elements such as LTRs, 5′ untranslated regions, and RRE; however, they have inactivating deletions in *pol*, *vif*, *vpr*, *vpu*, and *env*. To generate GagCeFP-MS2SL-CTE and GagCeFP-BglSL-CTE, a CTE-containing DNA fragment from B6-CTE was used to replace the corresponding RRE-containing DNA fragment in GagCeFP-MS2SL-RRE and GagCeFP-BglSL-RRE, respectively, using a two-step cloning process. The general structures of newly generated vectors were characterized by restriction enzyme mapping; inserted fragments that were amplified by PCR were verified by DNA sequencing.

### Cell Culture, Recombination Assay and Fusion Assay

The cell line 293T.CC, a kind gift from Dr. Robert Doms, is a derivative of the 293T cell line that expresses CD4 and CCR5. Hut/CCR5 and 293T cells were maintained as described previously [15]. Transfection of 293T cells was performed with the TransIT-LT1 reagent (Mirrus).

Cell lines containing two proviruses were produced as previously described [13] by sequential infection of viruses at low MOIs, followed by multiple rounds of cell sorting. To measure virus titers and recombination rates, viruses were harvested from producer cell lines transfected with the HIV-1 Env-expressing plasmid pIIINL(AD8) [37] and used to infect Hut/CCR5 target cells. Marker gene expression of target cells was detected by flow cytometry using cell staining with antibodies [13]. Infected cells were identified by expression of encoded markers (HSA, Thy, or B7), whereas cells infected with recombinant viruses were identified by the GFP^+^ phenotype using flow cytometry. The numbers of infected cells or cells infected with recombinants in the population were then converted to MOI using the Poison distribution. Recombination rates were calculated as the MOI of GFP^+^ cells divided by the MOI of infected cells. Additionally, infections were carried out at relatively low MOI when each parental viral titer was less than 0.5 and a minimum of 750 GFP^+^ cells were sampled in each measured rate.

To generate the cell lines used for the fusion assay, pseuodotyped viruses containing T6-NC* or H0-PTAP^–^ genomes were produced by cotransfection with helper plasmids expressing functional Gag. These viruses were used to infect 293T or 293T.CC cells separately; provirus-containing cells, identified by their expression of HSA or Thy marker, were enriched by two to three rounds of cell sorting. To perform the fusion assay, the provirus-containing 293T-based cell line was transfected with pIIINL(AD8). The provirus-containing 293T.CC-based cell line were cocultured with equal numbers of successfully transfected 293T cells at 24 h posttransfection. Viruses were harvested from the coculture 16 h after mixing the two cell lines, clarified through a 0.45-µm-pore-size filter, and used to infect Hut/CCR5 cells by spinnoculation (1200×g for 1 h at 15°C) in the presence of 10 µg/ml polybrene. Where indicated, the mixed cells were cultured in the presence of roscovitine, added at the time of mixing, at a final concentration of 10 µM.

### Single Virus Visualization

Human 293T cells were maintained as described above; transient transfection was performed with FuGENE HD (Roche) according to the manufacturer's recommendation. For all visualization experiments, 293T cells were cotransfected with a pair of modified HIV-1 plasmid DNA, one expressing GagCeFP and the other wild-type Gag at equal weight ratios; although only the plasmid with GagCeFP was mentioned for concise description. Virus particles were harvested 17 h posttransfection, and clarified by filtration as described above. To visualize particles, polybrene was added to the culture supernatant (25 µg/ml final concentration); this mixture was placed in a glass-bottom dish and incubated for 2 h at 37°C with 5% CO_2_ before image acquisition.

Epifluorescence microscopy was performed with an inverted Nikon TE2000E2 microscope and a 100×1.40 numerical aperture oil objective, using an X-Cite 120 system (EXFO Photonic Solution Inc.) for illumination. Digital images were acquired using a Hammamatsu ORCA-ER camera and Openlab software (Improvision) with the excitation and emission filter sets 427/10 nm and 472/30 nm for CeFP, 504/12 nm and 542/27 nm for YFP, and 577/25 nm and 632/60 nm for mCherry, respectively. Virus particle and RNA signal analysis and colocalization were performed by using custom software developed with Matlab™ and dipimage as previously described [21]. Merged images and pseudo-colored images were generated using ImageJ software.

## Supporting Information

Figure S1 Cell fusion without nuclear fusion. The cell line 293T.CC, expressing CD4 and CCR5, was mixed with 293T cells, which were transiently transfected with a plasmid encoding a CCR5 tropic HIV-1 Env. These two types of cells were mixed at equal ratios and were incubated for 16 hours in the presence of roscovitine prior to fixing. Prior to mixing the two cell types, the 293T cells were stained green with SYTO 11 (shown in panel A) and the 293TCC cells were stained red with SYTO 64 (shown in panel B). Nuclei were stained blue using Hoechst 33342 (panel C). Large, fused multinucleated cells appeared positive for both red and green (central large cell) and distinct nuclei (arrows, panel D) were observed in these fusion experiments demonstrating that these cells were fused but each nucleus remained intact at 16 hours.
(2.33 MB PDF)

Figure S2 Representative flow cytometry analysis of cells infected with the HIV-1 vector BIG reveals efficient co-expression of B7 and GFP markers. BIG is identical to B6-RRE (shown in Fig. 1) except it contains a functional *gfp* gene. The two marker genes, *B7* and *gfp*, are located in the *nef* reading frame; the translation of *gfp* is directed by an IRES.
(0.29 MB PDF)

Figure S3 Representative images of RNAs in single virions. In these images, Gag is detected with the CeFP signal, BglSL-containing HIV-1 RNA is detected with the mCherry signal, and MS2SL-containing HIV-1 RNA is detected with the YFP signal. Viral particles generated from cotransfection of GagCeFP-MS2SL-CTE and MS2-YFP (A), GagCeFP-MS2SL-CTE, GagCeFP-BglSL-CTE, MS2-YFP and Bgl-mCherry (B), and GagCeFP-MS2SL-CTE, GagCeFP-BglSL-RRE, MS2-YFP, and Bgl-mCherry (C). The channels used to detect these images are shown on top of each panel. To better demonstrate colocalization of signals, a merged and shifted panel was generated for each set of the images; in this panel, images captured from different channels were merged and the signals from the YFP channel were shifted by 4 pixels and signals from the mCherry channel were shifted by 8 pixels.
(0.19 MB PDF)

## References

[1] Hammarskjold ML (1997). Regulation of retroviral RNA export.. Semin Cell Dev Biol.

[2] Swanson CM, Malim MH (2006). Retrovirus RNA trafficking: from chromatin to invasive genomes.. Traffic.

[3] Rodriguez MS, Dargemont C, Stutz F (2004). Nuclear export of RNA.. Biol Cell.

[4] Bray M, Prasad S, Dubay JW, Hunter E, Jeang KT (1994). A small element from the Mason-Pfizer monkey virus genome makes human immunodeficiency virus type 1 expression and replication Rev-independent.. Proc Natl Acad Sci U S A.

[5] Fornerod M, Ohno M, Yoshida M, Mattaj IW (1997). CRM1 is an export receptor for leucine-rich nuclear export signals.. Cell.

[6] Neville M, Stutz F, Lee L, Davis LI, Rosbash M (1997). The importin-beta family member Crm1p bridges the interaction between Rev and the nuclear pore complex during nuclear export.. Curr Biol.

[7] Paca RE, Ogert RA, Hibbert CS, Izaurralde E, Beemon KL (2000). Rous sarcoma virus DR posttranscriptional elements use a novel RNA export pathway.. J Virol.

[8] Swanson CM, Puffer BA, Ahmad KM, Doms RW, Malim MH (2004). Retroviral mRNA nuclear export elements regulate protein function and virion assembly.. Embo J.

[9] Sherer NM, Swanson CM, Papaioannou S, Malim MH (2009). Matrix Mediates the Functional Link between Hiv-1 Rna Nuclear Export Elements and Gag Assembly Competency in Murine Cells.. J Virol.

[10] Hu WS, Temin HM (1990). Genetic consequences of packaging two RNA genomes in one retroviral particle: pseudodiploidy and high rate of genetic recombination.. Proc Natl Acad Sci U S A.

[11] Coffin JM (1979). Structure, replication, and recombination of retrovirus genomes: some unifying hypotheses.. J Gen Virol.

[12] Rhodes T, Wargo H, Hu WS (2003). High rates of human immunodeficiency virus type 1 recombination: near-random segregation of markers one kilobase apart in one round of viral replication.. J Virol.

[13] Rhodes TD, Nikolaitchik O, Chen J, Powell D, Hu WS (2005). Genetic recombination of human immunodeficiency virus type 1 in one round of viral replication: effects of genetic distance, target cells, accessory genes, and lack of high negative interference in crossover events.. J Virol.

[14] Chin MP, Rhodes TD, Chen J, Fu W, Hu WS (2005). Identification of a major restriction in HIV-1 intersubtype recombination.. Proc Natl Acad Sci U S A.

[15] Moore MD, Fu W, Nikolaitchik O, Chen J, Ptak RG (2007). Dimer initiation signal of human immunodeficiency virus type 1: its role in partner selection during RNA copackaging and its effects on recombination.. J Virol.

[16] Gorelick RJ, Gagliardi TD, Bosche WJ, Wiltrout TA, Coren LV (1999). Strict conservation of the retroviral nucleocapsid protein zinc finger is strongly influenced by its role in viral infection processes: characterization of HIV-1 particles containing mutant nucleocapsid zinc-coordinating sequences.. Virology.

[17] Huang M, Orenstein JM, Martin MA, Freed EO (1995). p6Gag is required for particle production from full-length human immunodeficiency virus type 1 molecular clones expressing protease.. J Virol.

[18] Boyko V, Leavitt M, Gorelick R, Fu W, Nikolaitchik O (2006). Coassembly and complementation of Gag proteins from HIV-1 and HIV-2, two distinct human pathogens.. Mol Cell.

[19] Castedo M, Roumier T, Blanco J, Ferri KF, Barretina J (2002). Sequential involvement of Cdk1, mTOR and p53 in apoptosis induced by the HIV-1 envelope.. Embo J.

[20] Wodrich H, Schambach A, Krausslich HG (2000). Multiple copies of the Mason-Pfizer monkey virus constitutive RNA transport element lead to enhanced HIV-1 Gag expression in a context-dependent manner.. Nucleic Acids Res.

[21] Chen J, Nikolaitchik O, Singh J, Wright A, Bencsics CE (2009). High efficiency of HIV-1 genomic RNA packaging and heterozygote formation revealed by Single virion analysis.. PNAS in press.

[22] Larson DR, Johnson MC, Webb WW, Vogt VM (2005). Visualization of retrovirus budding with correlated light and electron microscopy.. Proc Natl Acad Sci U S A.

[23] Jouvenet N, Bieniasz PD, Simon SM (2008). Imaging the biogenesis of individual HIV-1 virions in live cells.. Nature.

[24] Nikolaitchik O, Rhodes TD, Ott D, Hu WS (2006). Effects of mutations in the human immunodeficiency virus type 1 Gag gene on RNA packaging and recombination.. J Virol.

[25] Kharytonchyk SA, Kireyeva AI, Osipovich AB, Fomin IK (2005). Evidence for preferential copackaging of Moloney murine leukemia virus genomic RNAs transcribed in the same chromosomal site.. Retrovirology.

[26] Flynn JA, Telesnitsky A (2006). Two distinct Moloney murine leukemia virus RNAs produced from a single locus dimerize at random.. Virology.

[27] Rasmussen SV, Pedersen FS (2006). Co-localization of gammaretroviral RNAs at their transcription site favours co-packaging.. J Gen Virol.

[28] Anderson JA, Bowman EH, Hu WS (1998). Retroviral recombination rates do not increase linearly with marker distance and are limited by the size of the recombining subpopulation.. J Virol.

[29] Delviks KA, Pathak VK (1999). Effect of distance between homologous sequences and 3′ homology on the frequency of retroviral reverse transcriptase template switching.. J Virol.

[30] Nikolenko GN, Svarovskaia ES, Delviks KA, Pathak VK (2004). Antiretroviral drug resistance mutations in human immunodeficiency virus type 1 reverse transcriptase increase template-switching frequency.. J Virol.

[31] Onafuwa A, An W, Robson ND, Telesnitsky A (2003). Human immunodeficiency virus type 1 genetic recombination is more frequent than that of Moloney murine leukemia virus despite similar template switching rates.. J Virol.

[32] Levin JG, Rosenak MJ (1976). Synthesis of murine leukemia virus proteins associated with virions assembled in actinomycin D-treated cells: evidence for persistence of viral messenger RNA.. Proc Natl Acad Sci U S A.

[33] Butsch M, Boris-Lawrie K (2000). Translation is not required To generate virion precursor RNA in human immunodeficiency virus type 1-infected T cells.. J Virol.

[34] Dorman N, Lever A (2000). Comparison of viral genomic RNA sorting mechanisms in human immunodeficiency virus type 1 (HIV-1), HIV-2, and Moloney murine leukemia virus.. J Virol.

[35] Brandt S, Blissenbach M, Grewe B, Konietzny R, Grunwald T (2007). Rev proteins of human and simian immunodeficiency virus enhance RNA encapsidation.. PLoS Pathog.

[36] Fusco D, Accornero N, Lavoie B, Shenoy SM, Blanchard JM (2003). Single mRNA molecules demonstrate probabilistic movement in living mammalian cells.. Curr Biol.

[37] Freed EO, Martin MA (1995). Virion incorporation of envelope glycoproteins with long but not short cytoplasmic tails is blocked by specific, single amino acid substitutions in the human immunodeficiency virus type 1 matrix.. J Virol.

