# Supplementary figures and images for: Probing the HIV-1 Genomic RNA Trafficking Pathway and Dimerization by Genetic Recombination and Single Virion Analyses
Source: PLoS Pathog. 2009 Oct 16;5(10):e1000627. doi: 10.1371/journal.ppat.1000627 (PMC2757677; doi:10.1371/journal.ppat.1000627)

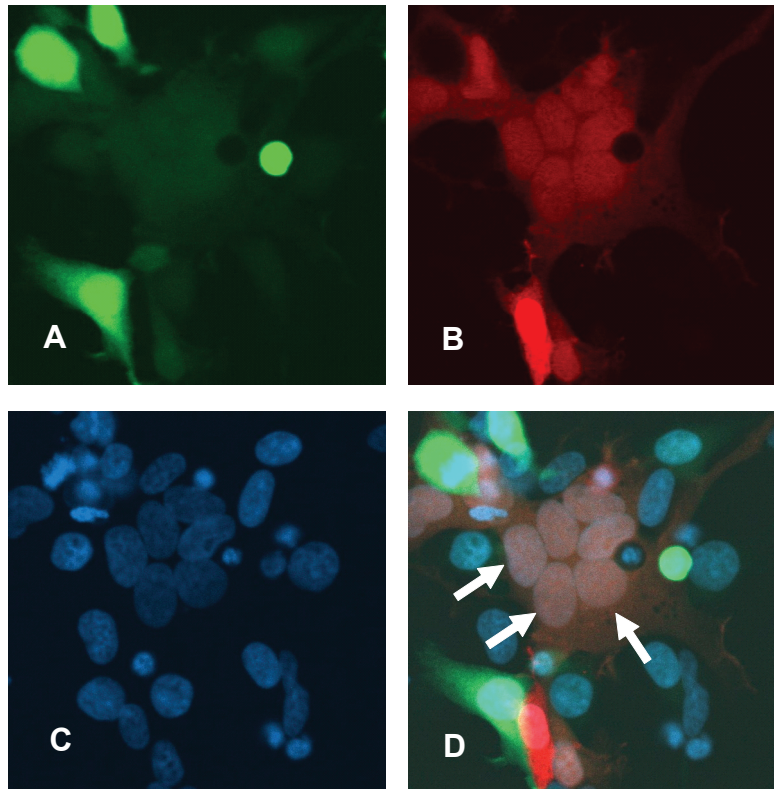

Moore et al Supplemental Figure 1

Supplement: Figure S1 — Cell fusion without nuclear fusion. The cell line 293T.CC, expressing CD4 and CCR5, was mixed with 293T cells, which were transiently transfected with a plasmid encoding a CCR5 tropic HIV-1 Env. These two types of cells were mixed at equal ratios and were incubated for 16 hours in the presence of roscovitine prior to fixing. Prior to mixing the two cell types, the 293T cells were stained green with SYTO 11 (shown in panel A) and the 293TCC cells were stained red with SYTO 64 (shown in panel B). Nuclei were stained blue using Hoechst 33342 (panel C). Large, fused multinucleated cells appeared positive for both red and green (central large cell) and distinct nuclei (arrows, panel D) were observed in these fusion experiments demonstrating that these cells were fused but each nucleus remained intact at 16 hours. (2.33 MB PDF) [file ppat.1000627.s001.pdf]

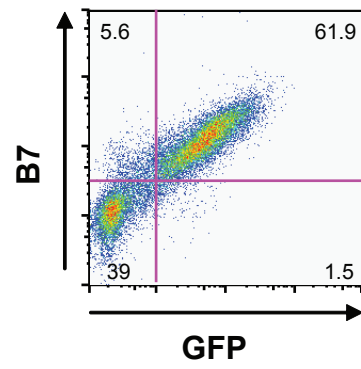

**Moore et al Supplemental Figure 2**

Supplement: Figure S2 — Representative flow cytometry analysis of cells infected with the HIV-1 vector BIG reveals efficient co-expression of B7 and GFP markers. BIG is identical to B6-RRE (shown in Fig. 1) except it contains a functional gfp gene. The two marker genes, B7 and gfp, are located in the nef reading frame; the translation of gfp is directed by an IRES. (0.29 MB PDF) [file ppat.1000627.s002.pdf]
