# Supplementary material for: Probing the HIV-1 Genomic RNA Trafficking Pathway and Dimerization by Genetic Recombination and Single Virion Analyses
Source: PLoS Pathog. 2009 Oct 16;5(10):e1000627. doi: 10.1371/journal.ppat.1000627 (PMC2757677; doi:10.1371/journal.ppat.1000627)
Supplement: Figure S3 — Representative images of RNAs in single virions. In these images, Gag is detected with the CeFP signal, BglSL-containing HIV-1 RNA is detected with the mCherry signal, and MS2SL-containing HIV-1 RNA is detected with the YFP signal. Viral particles generated from cotransfection of GagCeFP-MS2SL-CTE and MS2-YFP (A), GagCeFP-MS2SL-CTE, GagCeFP-BglSL-CTE, MS2-YFP and Bgl-mCherry (B), and GagCeFP-MS2SL-CTE, GagCeFP-BglSL-RRE, MS2-YFP, and Bgl-mCherry (C). The channels used to detect these images are shown on top of each panel. To better demonstrate colocalization of signals, a merged and shifted panel was generated for each set of the images; in this panel, images captured from different channels were merged and the signals from the YFP channel were shifted by 4 pixels and signals from the mCherry channel were shifted by 8 pixels. (0.19 MB PDF) [file ppat.1000627.s003.pdf]

A

GagCeFP-MS2SL-CTE + MS2-YFP

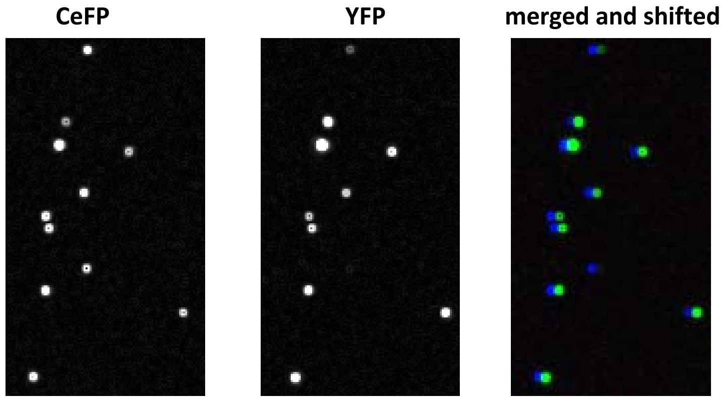

B

GagCeFP-MS2SL-CTE + GagCeFPBglSL-CTE + MS2-YFP + Bgl-mCherry

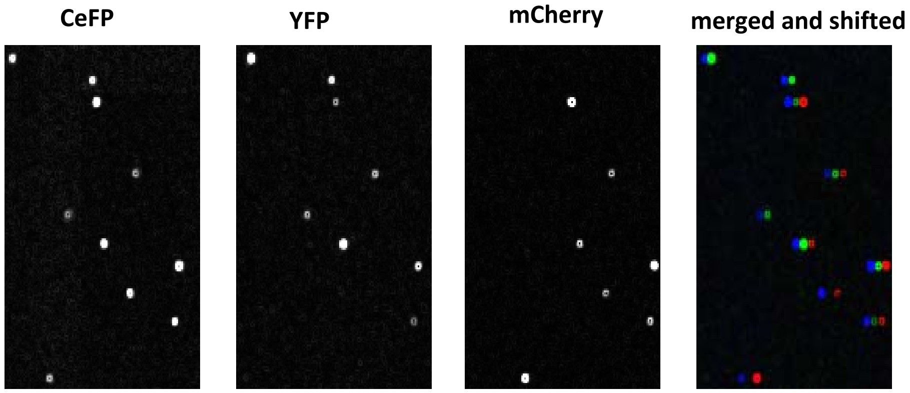

C

GagCeFP-MS2SL-CTE + GagCeFP-BglSL-RRE + MS2-YFP + Bgl-mCherry

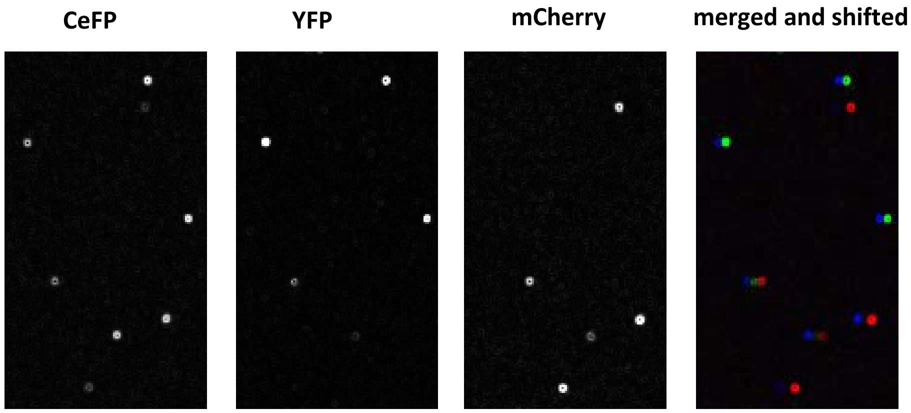

Moore et al Supplemental Figure 3
